# First complete mitochondrial genome of the endemic goby, *Rhinogobius davidi* (Gobiiformes: Gobiidae: Gobionellinae), in China

**DOI:** 10.1080/23802359.2023.2189493

**Published:** 2023-03-14

**Authors:** Lin Song, Xiao Jiang Chen, Yang Song, Quan Wang

**Affiliations:** Jiangsu Agri-animal Husbandry Vocational College, Taizhou, Jiangsu Province, P. R.China

**Keywords:** Mitochondrial genome, *Rhinogobius davidi*, Gobionellinae, phylogenetic analysis

## Abstract

The first complete mitochondrial genome of the freshwater goby *Rhinogobius davidi* was determined by high-throughput sequencing. This genome was 16,627 bp in length and consisted of 13 protein-coding genes, 22 transfer RNA genes, 2 ribosomal RNA genes, and 2 non-coding control regions. Phylogenetic analysis based on the amino acid sequences of 13 mitochondrial protein-coding genes from *R. davidi* and 23 relatives suggested that *R. davidi* had a close mitogenome relationship with *Rhinogobius giurinus*. This complete genome of *R. davidi* will provide basal molecular data for future studies on taxonomy, comparative genomics, and adaptive evolution in *Rhinogobius*.

## Introduction

**Figure 1.**
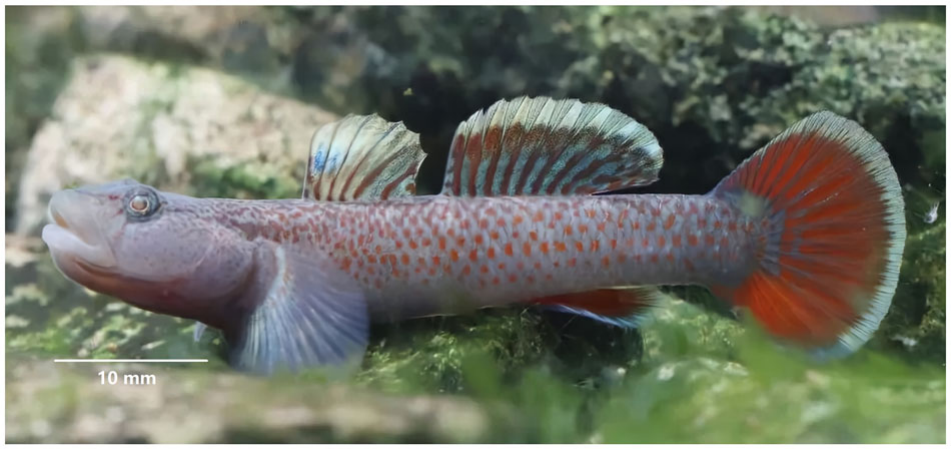
The specimen of *Rhinogobius davidi* from Cao’e River, Shaoxing, Zhejiang Province, China (Photo by Lin Song).

*Rhinogobius davidi* (Sauvage & Dabry de Thiersant, 1874) is a benthic species of landlocked goby distributed in the Oujiang, Qiantang, and Yangtze River drainages, China (Figure 1). This fish feeds on invertebrates and appears to be limited to smaller streams in the upper reaches of these large river drainages. There are no records of the species occurring in hydrostatic environments such as lakes in these drainages. The breeding period begins when the water temperature is above 20 °C. Before breeding, the male fish nests, and then attracts the females to enter and lay eggs. The eggs adhere to the ventral surface of the stones, and the male protects the eggs alone until they hatch. Female fish can reproduce once a month, each spawning up to 100 or more (Li 2011). Distinguishing morphological features of this species include: first dorsal fin VI, second dorsal fin I, 9–10, gluteal fin I, 6–8, pectoral fin 14–15, ventral fin I, 5; longitudinal scales 30–32, transverse scales 11, dorsal fin anterior scales 0–2 (mainly 0); vertebrae number 11 + 17 = 28; no tube hole in the sensory tube of the anterior gill cover; cheek with a brown vertical stripe below the eye (Yang et al. 2008; Li 2011).

*Rhinogobius davidi* is of ornamental value, but to date, it is poorly studied and what research has been done is limited to morphology and ecology (Chen and Miller 1998; Li 2011). Due to various manmade influences, the ecological environment of the basin is seriously damaged and the number of fish declined significantly. Genetic information is crucial to the development of strategies for the identification and management of this species (Yang et al. 2020). Therefore, this study is aimed to provide a first report on the mitochondrial genome of *R. davidi* and analyze the phylogenetic relationships among the genus *Rhinogobius*.

## Materials

The specimens were obtained from Cao’e River in Shangyu District, Shaoxing, Zhejiang Province of China (29°57′17.94″N, 120°52′06.98″E), and were identified as *R. davidi* based on the morphological characters. Some specimens were kept in dry ice until DNA analysis and transferred to Shanghai Genesky Biotechnologies Inc, while others (Voucher number ASTIH-21b1108d23) were fixed in 95% ethanol and deposited in Aquatic Science and Technology Institution Herbarium (https://www.jsahvc.edu.cn/) with Lin Song (tianxinlinger@126.com) in charge. All animal handling and experimental procedures were performed in accordance with the recommendations of the Ethics Committee for Animal Experiments of Jiangsu Agri-animal Husbandry Vocational College (Taizhou, China).

## Methods

The total genomic DNA was extracted from muscle tissue using the phenol-chloroform method (Barnett and Larson 2012). The DNA library was established with quality-controlled DNA samples, and amplified by high-fidelity polymerase to ensure sufficient library volume on the sequencer. Agilent 2100 Bioanalyzer (Agilent Technologies, USA) was applied to determine the size distribution of library fragments and evaluate the suitability for sequencing. After library pooling, next-generation sequencing was performed on Illumina HiSeq 4000 Sequencing platform (Illumina, CA, USA). The raw sequencing data were checked by FastQC, and removed of low-quality reads and adapter region with Trimmomatic (Bolger et al. 2014). The trimmed reads were mapped to the reference mitogenome of *Rhinogobius giurinus* (KU871066), using BWA v.0.7.17 (Li and Durbin 2009) with default parameters.

Samtool v.1.9 (Li and Durbin 2009) was employed to retrieve the aligned mitochondrial reads. The aligned reads with the mitogenome were then assembled using the software MetaSPAdes 3.13.0 (Nurk et al. 2017).

The resulting contig was annotated on MitoMaker 1.14 (Bernt et al. 2013). The final full mtDNA sequence is available in GenBank under accession number OM617724. And the genome circular map of *R. davidi* was drawn by CGView online server (https://proksee.ca/) (Grant and Stothard 2008).

We generated a phylogenetic tree with a set of all 22 *Rhinogobius* species available in GenBank and 2 *Chaenogobius* species. Each of the 13 PCGs was aligned separately using MUSCLE algorithm (Edgar 2004) in MEGA X, then the 13 PCG alignments were concatenated into a single multiple sequence alignment. The substitution model mtREV + G + I + F was selected utilizing the Find Best DNA/protein model tool of MEGA X software. The maximum likelihood analysis was performed on MEGA X with 1000 bootstrap replicates (Kumar et al. 2018). *Chaenogobius gulosus* (Oh et al. 2016) and *Chaenogobius annularis* were selected for the outgroups.

## Results

**Figure 2.**
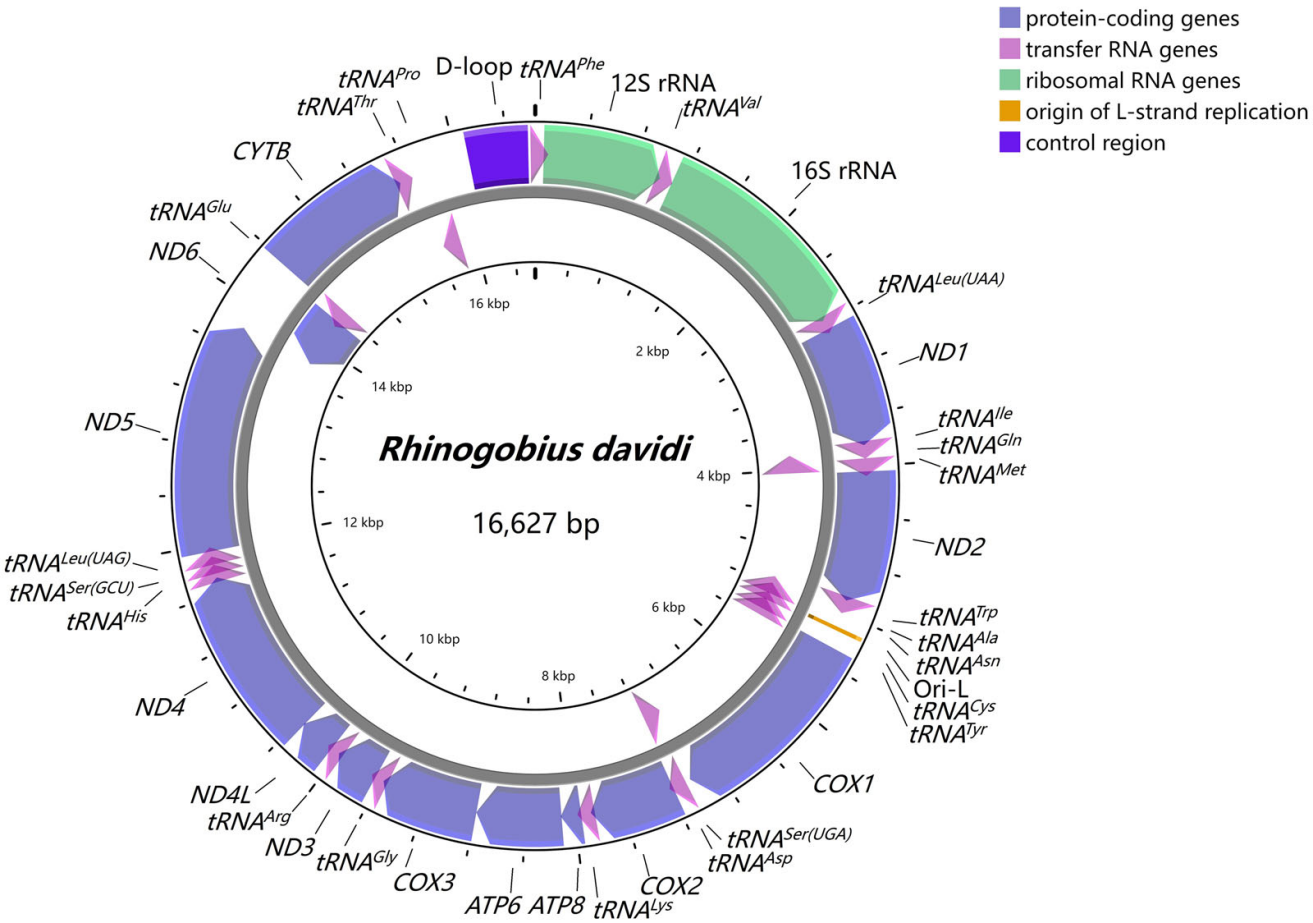
Gene map of the mitochondrial genome of *Rhinogobius davidi*. Gene encoded on H- and L-strands with inverse arrow directions were shown outside and inside the circle, respectively. The complete mitogenome of *R. davidi* is 16,627 bp with the inclusion of 13 protein-coding genes, 22 transfer RNA genes, 2 ribosomal RNA genes, origin of L-strand replication (Ori-L) and control region (D-loop).

The complete mitochondrial genome of *R. davidi* comprised 13 protein-coding genes (PCGs), 22 transfer RNA genes (tRNAs), 2 ribosomal RNA genes (12S rRNA and 16S rRNA), and 2 non-coding control regions (control region and origin of light-strand replication), which formed a double-stranded circular molecule of 16,627 bp in length (Figure 2). All other genes were encoded on the heavy strand (H-strand), except for one protein-coding gene (*ND6*) and eight tRNA genes (*tRNA^Gln^*, *tRNA^Ala^*, *tRNA^Asn^*, *tRNA^Cys^*, *tRNA^Tyr^*, *tRNA^Ser(UCN)^*, *tRNA^Glu^*, and *tRNA^Pro^*). The overall nucleotide composition in descending order was 17% for G, 25.9% for T, 27% for A, and 30.1% for C, with a slight AT-rich feature (52.9%).

Twelve protein-coding genes began with the standard ATG, whereas *COX1* initiated with GTG. There were both complete termination codons (TAA for *ND1*, *COX1*, *ATP8*, *ATP6*, *ND4L*, *ND5*; TAG for *ND2*, *ND3*, *ND6*) and incomplete termination codons (TA for *COX3*, T for *COX2*, *ND4*, *CYTB*). Four overlapping regions revealed between protein-coding genes were *ATP8-ATP6*, *ATP6-COX3*, *ND4L-ND4*, and *ND5-ND6*, and there were two overlaps between tRNAs (*tRNA^Ile^*-*tRNA^Gln^*, *tRNA^Gln^*-*tRNA^Met^*). For rRNAs, 12S rRNA was located between *tRNA^Phe^* and *tRNA^Val^* with 954 bp in length, and 16S rRNA was located between *tRNA^Val^* and *tRNA^Leu(UUR)^* with 1,661 bp in length. They were separated by *tRNA^Val^* with the same situation found in other gobies (Wang et al. 2019; Tan et al. 2020). 22 tRNAs ranged from 65 to 76 bp, which were conservative and scattered throughout the mitogenome of *R. davidi.* The control region, which was 476 bp long, was located between *tRNA^Phe^* and *tRNA^Pro^*.

**Figure 3.**
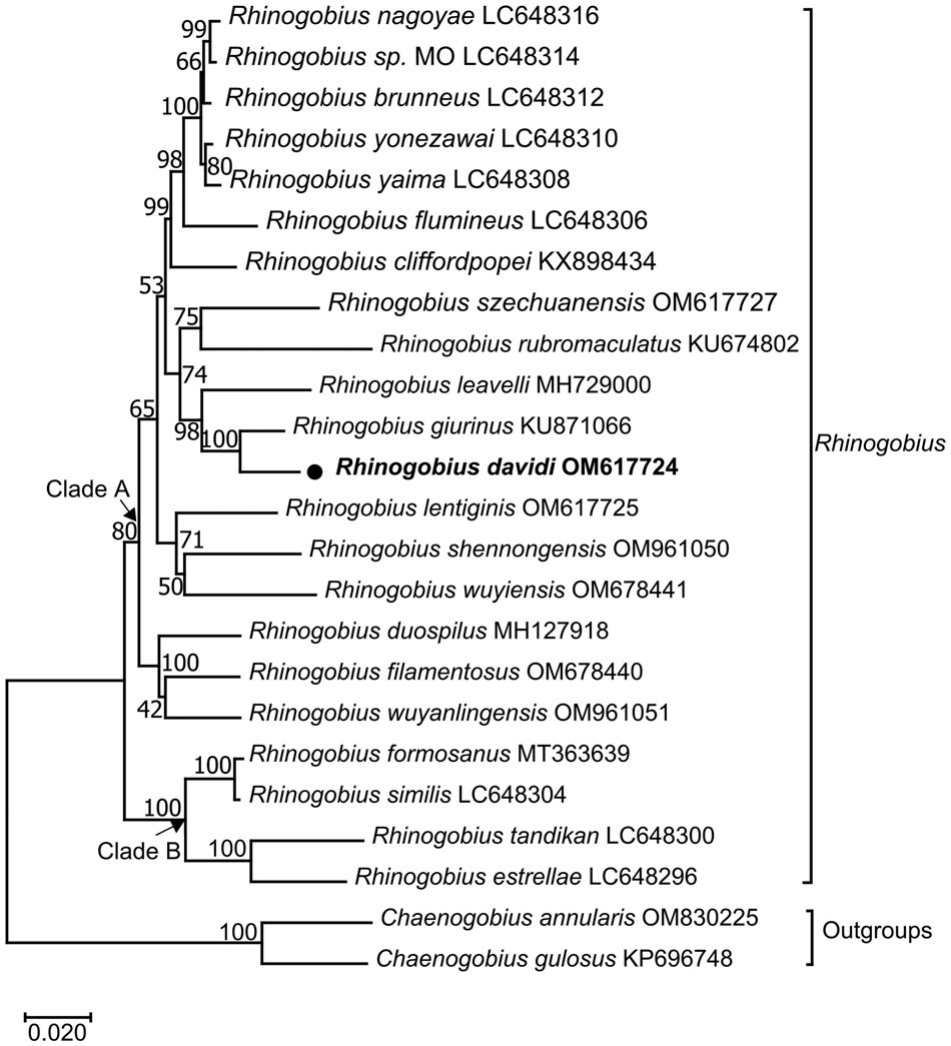
Maximum-likelihood (ML) phylogenetic tree from amino acid sequences of 13 PCGs of *Rhinogobius davidi* and other 23 fishes. Accession numbers were indicated after the species names. The tree topology was evaluated by 1000 bootstrap replicates. Bootstrap values at the nodes correspond to the support values for ML methods. The tree was drawn to scale, with branch lengths measured in the number of substitutions per site.

The maximum-likelihood phylogenetic tree showed the mitochondrial genome relationship of *R. davidi* in *Rhinogobius.* In Figure 3, R*. davidi* clustered into a clade with *R. giurinus* (Xie et al. 2015), and then with *R. leavelli* (Zhang and Shen 2019). The three taxa, together with nine other *Rhinogobius* species (*R. nagoyae* (Maeda et al. 2021), *R. sp. MO* (Maeda et al. 2021), *R. brunneus* (Maeda et al. 2021), *R. yonezawai* (Maeda et al. 2021), *R. yaima* (Maeda et al. 2021), *R. flumineus* (Maeda et al. 2021), *R. cliffordpopei* (Wang et al. 2019), *R. szechuanensis*, *R. rubromaculatus*), constituted a sister-group to *R. lentiginis, R. shennongensis* and *R. wuyiensis*. All the above 15 *Rhinogobius* species formed Clade A with *Rhinogobius duospilus* (Tan et al. 2020), *Rhinogobius filamentosus* (Chen et al. 2022) and *Rhinogobius wuyanlingensis* (Song et al. 2022). In addition, *Rhinogobius formosanus* (Yang et al. 2020), *Rhinogobius similis* (Maeda et al. 2021), *Rhinogobius estrellae* (Maeda et al. 2021) and *Rhinogobius tandikan* (Maeda et al. 2021) formed Clade B, which was sister to Clade A.

## Discussion and conclusion

We reported the first complete mitogenome sequencing of *R. davidi* by high-throughput sequencing and assembly. The arrangement and orientation of all 37 genes were in accordance with those goby mitogenomes published previously (Xie et al. 2015; Wang et al. 2019). In this study, more molecular information from mitogenomes of *Rhinogobius* was used to reconstruct the phylogenetic mitochondrial genome relationships, and the ML tree placed *R. davidi* in a well-supported cluster with *Rhinogobius giurinus.* The overall topology was similar to those of previous studies (Zhang and Shen 2019; Tan et al. 2020). The results presented here will be essential to the specimen identification, fishery resources management, and further phylogenetic studies on Gobionellinae species.

## Supplementary Material

Supplemental Material

Supplemental Material

Supplemental Material

Supplemental Material

## Data Availability

The genome sequence data that support the findings of this study are openly available in GenBank of NCBI at (https://www.ncbi.nlm.nih.gov/) under the reference number OM617724. The associated “BioProject", “Bio-Sample” and “SRA” numbers are PRJNA808178, SAMN26030965, and SRR18064556 respectively.
