# Supplementary figures and images for: First complete mitochondrial genome of the endemic goby, Rhinogobius davidi (Gobiiformes: Gobiidae: Gobionellinae), in China
Source: Mitochondrial DNA B Resour. 2023 Mar 14;8(3):410–3. doi: 10.1080/23802359.2023.2189493 (PMC10026808; doi:10.1080/23802359.2023.2189493)

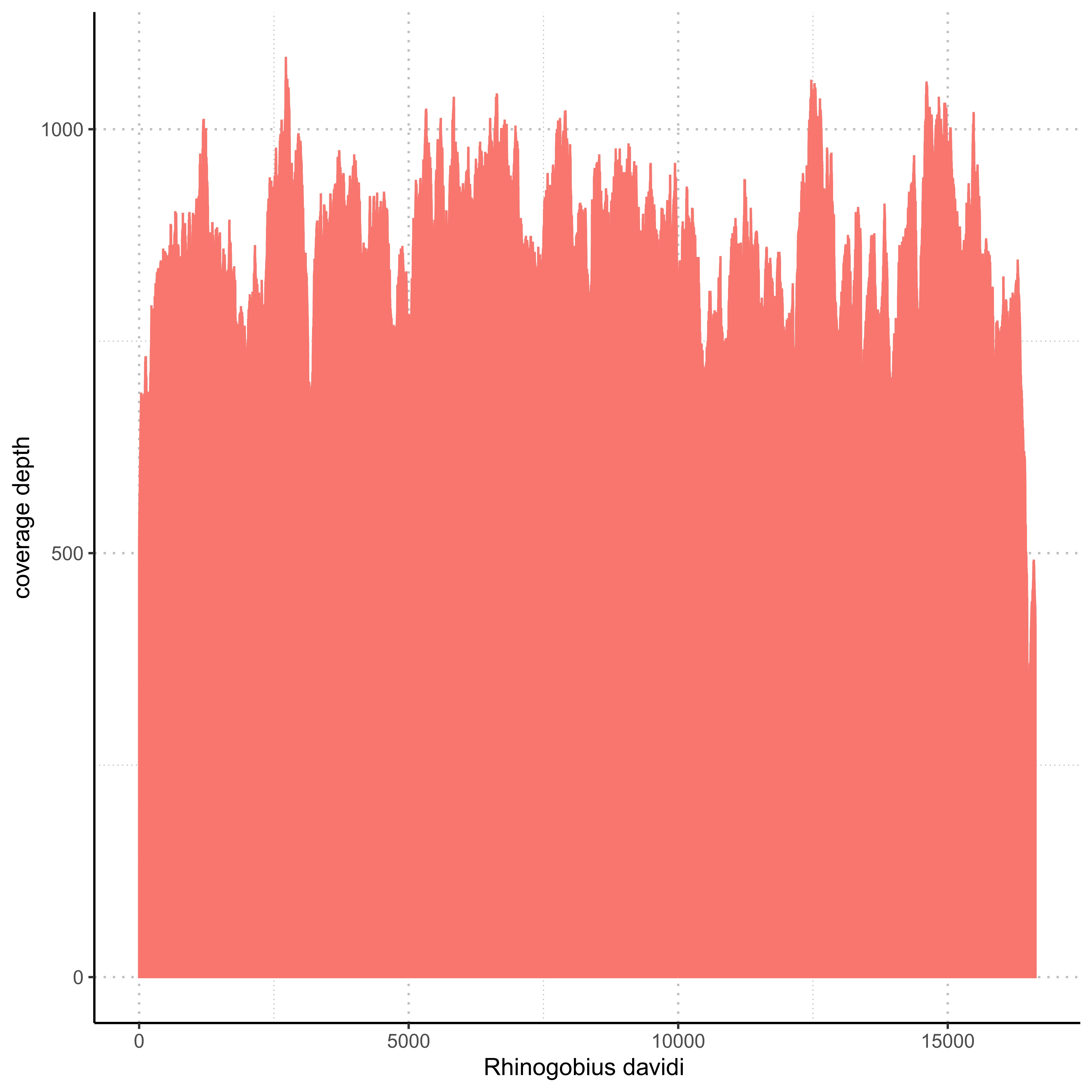

Supplement: Supplemental Material [file TMDN_A_2189493_SM0295.jpg]
